# Predictors of physical restraint use in Canadian intensive care units

**DOI:** 10.1186/cc13789

**Published:** 2014-03-24

**Authors:** Elena Luk, Barbara Sneyers, Louise Rose, Marc M Perreault, David R Williamson, Sangeeta Mehta, Deborah J Cook, Stephanie C Lapinsky, Lisa Burry

**Affiliations:** 1 Lawrence S. Bloomberg Faculty of Nursing, University of Toronto, 155 College Street, Rm 276, Toronto, ON M5T 1P8, Canada; 2 Louvain Drug Research Institute, Université catholique de Louvain, Avenue Hippocrate, 10, Brussels, Belgium; 3 Faculté de pharmacie, Université de Montréal, CP 6128 succursale centre-ville, Montréal, QC H3C 3J7, Canada; 4 Mount Sinai Hospital, 600 University Avenue, Toronto, ON M5G 1X5, Canada; 5 Departments of Medicine, Clinical Epidemiology and Biostatistics, McMaster University Health Sciences Center, 1200 Main Street West, Hamilton, ON L8N 3Z5, Canada; 6 Faculty of Medicine, University of Toronto, 1 King’s College Circle, Medical Sciences Building, Toronto, ON M5S 1A8, Canada

## Abstract

**Introduction:**

Physical restraint (PR) use in the intensive care unit (ICU) has been associated with higher rates of self-extubation and prolonged ICU length of stay. Our objectives were to describe patterns and predictors of PR use.

**Methods:**

We conducted a secondary analysis of a prospective observational study of analgosedation, antipsychotic, neuromuscular blocker, and PR practices in 51 Canadian ICUs. Data were collected prospectively for all mechanically ventilated adults admitted during a two-week period. We tested for patient, treatment, and hospital characteristics that were associated with PR use and number of days of use, using logistic and Poisson regression respectively.

**Results:**

PR was used on 374 out of 711 (53%) patients, for a mean number of 4.1 (standard deviation (SD) 4.0) days. Treatment characteristics associated with PR were higher daily benzodiazepine dose (odds ratio (OR) 1.05, 95% confidence interval (CI) 1.00 to 1.11), higher daily opioid dose (OR 1.04, 95% CI 1.01 to 1.06), antipsychotic drugs (OR 3.09, 95% CI 1.74 to 5.48), agitation (Sedation-Agitation Scale (SAS) >4) (OR 3.73, 95% CI 1.50 to 9.29), and sedation administration method (continuous and bolus versus bolus only) (OR 3.09, 95% CI 1.74 to 5.48). Hospital characteristics associated with PR indicated patients were less likely to be restrained in ICUs from university-affiliated hospitals (OR 0.32, 95% CI 0.17 to 0.61). Mainly treatment characteristics were associated with more days of PR, including: higher daily benzodiazepine dose (incidence rate ratio (IRR) 1.07, 95% CI 1.01 to 1.13), daily sedation interruption (IRR 3.44, 95% CI 1.48 to 8.10), antipsychotic drugs (IRR 15.67, 95% CI 6.62 to 37.12), SAS <3 (IRR 2.62, 95% CI 1.08 to 6.35), and any adverse event including accidental device removal (IRR 8.27, 95% CI 2.07 to 33.08). Patient characteristics (age, gender, Acute Physiology and Chronic Health Evaluation II score, admission category, prior substance abuse, prior psychotropic medication, pre-existing psychiatric condition or dementia) were not associated with PR use or number of days used.

**Conclusions:**

PR was used in half of the patients in these 51 ICUs. Treatment characteristics predominantly predicted PR use, as opposed to patient or hospital/ICU characteristics. Use of sedative, analgesic, and antipsychotic drugs, agitation, heavy sedation, and occurrence of an adverse event predicted PR use or number of days used.

## Introduction

Physical restraint (PR) is applied in the intensive care unit (ICU) to prevent unplanned treatment interference that can lead to serious patient harm such as self-extubation. PR use in the ICU is controversial because restraints may present an ethical dilemma, conflicting with values of humane and respectful care; furthermore, PR can be perceived as barbaric, cruel, and obstructing patient autonomy [1-5]. PR has been linked to undesirable patient outcomes including delirium, post-traumatic stress disorder, higher rates of self-extubation, and prolonged ICU length of stay [6-8].

International descriptions of prevalence of physically restrained ICU patients vary from 0% to 100% across different countries [9-13]. Numerous policy and guideline documents aim to minimize PR practice variability and use [14-18], such as Canada’s province of Ontario’s *Patient Restraints Minimization Act*, which legislates restraint reduction to maintain patient safety [16]. Unfortunately, few evidence-based recommendations on methods to minimize restraint use are available due to the limited number and poor quality of existing studies. Most studies describe the prevalence, and reasons and context for PR use, but do not identify modifiable predictors [9-13,19-22].

We conducted a secondary analysis of PR use in a large, heterogeneous sample of mechanically ventilated (MV) patients admitted to 51 Canadian ICUs [23]. Our objectives were to: (1) describe patterns of PR use in MV patients (prevalence, number of days of use, number of episodes of use); and (2) identify patient, treatment, and ICU/hospital characteristics associated with PR use and number of days of use.

Previous studies from geriatric and nursing home settings showed non-modifiable patient characteristics, such as cognitive impairment, may influence PR use [24,25]. Therefore, we anticipated the latter might predict PR use in the ICU. As international practice recommendations are to reduce excessive sedation [26], we hypothesized that sedation practices such as type or dose of sedative-analgesic or use of sedation minimization strategies might influence PR practices. Finally, we expected organizational characteristics might be associated with PR use, as suggested by survey data [9].

## Materials and methods

We conducted a secondary analysis of the I-CAN-SLEAP database. I-CAN-SLEAP was a prospective, observational study describing analgo-sedation, antipsychotic, and neuromuscular blocker administration and drug assessment or titration practices in 51 Canadian ICUs [23]. ICUs were recruited from all 10 provinces between 2008 and 2009, representing university-affiliated and community hospitals. Patients were included in each ICU during a predefined two-week period. Patient inclusion criteria were 1) initiation of MV during the inclusion period; and 2) age ≥16 years. Data were collected from initiation of MV until extubation, 24 hours after tracheotomy, death, or for a maximum of 30 days. Each site’s Research Ethics Board (REB) approved the research protocol and waived the need for informed consent. An additional file shows a list of all REBs that approved the study (see Additional file 1).

We collected site level data on hospital and ICU characteristics including province, hospital type (university-affiliated or community), number of beds (hospital and ICU), ICU type (for example, medical, surgical), physician model (open or closed; closed defined as patient care led by the ICU team), proportion of ventilator-capable beds in the ICU, and availability of protocols and assessment scales for sedation, analgesia, and delirium. Nurse-to-patient ratio was collected on each patient daily.

We collected data on baseline patient characteristics including age, gender, Acute Physiology and Chronic Health Evaluation (APACHE) II score [27], diagnosis, comorbidities, medication history, smoking, alcohol, and prior drug use. Additional daily patient data that we characterized as treatment characteristics included: PR use (yes or no); mode of MV; doses of sedative, analgesic, antipsychotic, and neuromuscular blocking drugs; presence of organ failure; mode of sedation administration (intermittent use vs. continuous infusion vs. both); daily sedation interruption (DSI); use of sedation protocols; use of sedation, pain and delirium assessment scales; and adverse events defined as deliberate or accidental device removal (endotracheal tube, intravenous catheter, feeding tube and urinary catheter) by patients or accidental removal by staff, and danger for self or others. Doses of opioids were converted to morphine equivalents, and those of benzodiazepines to midazolam equivalents [28]. All sedation scores were converted to Sedation-Agitation Scale (SAS) scores [29] and were classified *a priori* as: over-sedated (SAS <3), lightly sedated (SAS 3 to 4) and agitated (SAS >4). An additional file describes scale conversions and scoring definitions in more detail (see Additional file 2).

### Statistical analysis

PR prevalence was defined as PR use on at least one day during the study period. PR prevalence, demographic characteristics and clinical variables are presented as means and standard deviations (SD), and frequencies, proportions and 95% confidence intervals (CIs) for categorical variables. Demographic characteristics for ‘ever restrained’ and ‘never-restrained’ patients were compared using chi-square tests for categorical variables and Wilcoxon rank sum tests or two-sample *t* tests, depending on data distribution, for continuous variables.

Using multivariable logistic regression, we assessed patient, institutional and clinical factors associated with PR use at any time during the study period, and reported results using odds ratios (OR) and their associated 95% CIs. Using Poisson regression analysis, we examined associations between patient, institutional and clinical variables and the number of days of PR use, and reported incidence rate ratios (IRR) and their associated 95% CIs. Variables entered into each of the two models were selected *a priori* based on a review of the literature on restraint use in diverse populations. Prior to multivariable modeling, variables were assessed for multicollinearity using tolerance statistics. A tolerance value of <0.4 was used to indicate the presence of multicollinearity, which was not a concern in this analysis. The number of variables retained in the model was based on rules of modeling [30] and these rules were not violated for either logistic or Poisson model. All tests were two-tailed with a *P*-value ≤0.05 deemed significant. An independent statistician conducted all analyses using SAS 9.2 (SAS Institute, Cary, NC, USA).

## Results

PR was used on one or more days for 374/711 patients (53%, 95% CI 49% to 56%). Patients were restrained on an average of 4.1 (SD 4.0) days, with a range of 1 to 26 days. Most patients (83%, 311/374) were restrained only once, the remainder had restraints removed and reapplied more than once during their ICU admission. Restrained and never-restrained patients had similar baseline characteristics; however, differences in treatment characteristics were noted (Table 1). Restrained patients experienced more adverse events, received higher daily doses of benzodiazepines, propofol, and opioids, received more days of antipsychotics, experienced DSI more frequently, and were agitated (SAS >4) and over-sedated (SAS <3) on more days.

**Table 1:** Characteristics of patients who were restrained and never restrained

**Data point**^***a***^	**Non-restrained (n = 337)**	**Restrained (n = 374)**
**Patient characteristics**		
Age (years)	60.6 (16.6)	61.1 (16.8)
Gender (male)	212 (63)	230 (62)
APACHE II score	19.9 (8.0)	19.4 (7.7)
Patient admission category		
Medical	124 (37)	156 (42)
Surgical	115 (34)	132 (35)
Cardiac	52 (15)	33 (9)
Neurologic/trauma	35 (10)	41 (11)
Other	11 (3)	12 (3)
Duration of organ dysfunction (days)		
Renal failure^*b*^	0.9 (2.4)	1.4 (3.3)
Hepatic failure^*c*^	0.4 (1.6)	1.0 (3.2)
Inotrope/vasopressor support (days)^*d*^	1.4 (2.0)	1.9 (3.1)
Cognitive impairment (dementia)	7 (2)	9 (2)
Psychiatric condition^*e*^	45 (13)	53 (14)
Prior use of sedative, opioid, antidepressant	113 (34)	113 (30)
Prior use of antipsychotic	25 (7)	31 (8)
Current smokers	56 (17)	68 (18)
Alcohol consumption	80 (24)	100 (27)
Habitual drug use	18 (5)	15 (4)
**Treatment characteristics**		
Daily drug use		
Benzodiazepines (mg^*f*^)*	10.8 (34.0)	29.6 (65.8)
Propofol (mg)*	91.1 (523.5)	104.1 (501.2)
Opioids (mg^*g*^)*	32.9 (60.2)	64.6 (91.8)
Daily sedation interruption (days)*	0.7 (1.1)	1.2 (1.7)
Sedation-Agitation Scale scores (days)*		
Agitation (SAS >4)^h^*	0.1 (0.3)	0.4 (1.2)
Over-sedation (SAS <3)^h^*	1.1 (2.1)	1.9 (2.7)
Antipsychotic administration (days)*	0.2 (0.9)	1.2 (3.0)
Duration of mechanical ventilation (days)*	3.1 (3.5)	6.8 (6.5)
Occurrence of adverse event^i^*	9 (3)	24 (6)

^*a*^Values are n (%) for categorical variables and means (standard deviations) for continuous variables; ^*b*^renal failure was defined as creatinine clearance <30 ml/min, serum creatinine >180 μmol/L or need for dialysis; ^*c*^hepatic failure was defined as aspartate aminotransferase (AST) or alanine transaminase (ALT) >2 times the upper limit of normal or bilirubin >3 times the upper limit of normal; ^*d*^inotrope or vasopressor support: administration of inotropes and vasopressors at any dose; ^*e*^psychiatric condition included depression, anxiety, bipolar disorder, schizophrenia; ^*f*^dose expressed in midazolam equivalents (1 mg midazolam = 0.5 mg lorazepam); ^*g*^dose expressed in morphine equivalents (10 mg morphine = 2 mg hydromorphone = 0.1 mg fentanyl); ^*h*^Sedation-Agitation Scale; ^*i*^adverse events comprised deliberate or accidental device removal (endotracheal tube, intravenous lines, feeding tubes, urinary catheters) by patients or accidental removal by staff, and danger to self or others. *Difference between groups was statistically significant (*P* <0.05). APACHE II, Acute Physiology and Chronic Health Evaluation II.

PR was used on an average of 76% (95% CI 66% to 85%) of days the patients’ SAS was >4; and 58% (95% CI 51% to 65%) of days the patients’ SAS was <3. PR was used on an average of 42% (95% CI 34% to 50%) of days with DSI, 65% (95% CI 55% to 75%) of days an antipsychotic was prescribed, and 61% (95% CI 45% to 76%) of days an adverse event occurred.

Treatment variables independently associated with PR use comprised: higher benzodiazepine and opioid daily doses, sedation administration method (continuous and bolus vs. bolus only), ever receiving an antipsychotic, and ever scoring SAS >4 (Table 2). For every 10 mg increment in morphine-equivalent dose and for every 10 mg increment in midazolam-equivalent dose, the risk of PR increased by 4% and 5% respectively. PR use was less likely in university-affiliated hospitals. Patients were more likely to be restrained when the ICU proportion of ventilator-capable beds was >50% and ≤90% as compared to when the ICU proportion of ventilator-capable beds was <25%. Variables independently associated with more days of PR use included higher daily benzodiazepine dose, DSI, ever receiving an antipsychotic, SAS <3 and occurrence of an adverse event (Table 3). Patients were more likely to be restrained for more days in ICUs where proportion of ventilator-capable beds was 25 to 50% and 76 to 90% compared to ICUs where proportion of ventilator-capable beds was <25%.

**Table 2:** Factors independently associated with physical restraint use

**Data point**	**Univariable**	**Multivariable**
	**OR**^***a ***^**(95% CI)**	**OR**^***a ***^**(95% CI)**
**Patient characteristics**		
Age	1.00 (0.99-1.01)	1.00 (0.99-1.01)
Male sex	0.98 (0.71-1.33)	1.15 (0.78-1.69)
Psychiatric condition^*b*^	1.02 (0.66-1.56)	0.86 (0.48-1.55)
Cognitive impairment (dementia)	1.01 (0.36-2.81)	1.42 (0.40-5.00)
Prior psychotropic drug use^*c*^	0.91 (0.67-1.24)	0.98 (0.64-1.50)
Smoking or alcohol consumption, habitual drug use	0.99 (0.73-1.36)	1.03 (0.70-1.53)
Patient category		
Surgical	1	1
Medical	0.93 (0.66-1.32)	0.96 (0.60-1.52)
Other	0.75 (0.51-1.11)	0.96 (0.58-1.59)
APACHE II score	0.99 (0.97-1.01)	1.00 (0.97-1.02)
**Treatment characteristics**		
Medication use per mechanical ventilation days		
Benzodiazepines (10 mg increments^*d*^)	1.11 (1.06-1.16)	1.05 (1.00-1.11)
Propofol (10 mg increments)	1.00 (1.00-1.00)	1.00 (1.00-1.00)
Opioids (10 mg increments^*e*^)	1.06 (1.04-1.09)	1.04 (1.01-1.06)
Daily sedation interruption	1.36 (1.00-1.84)	1.46 (0.93-2.30)
Sedation administration		
Intermittent use only	1	1
Continuous infusion only	1.43 (0.93-2.21)	1.39 (0.74-2.59)
Both	4.14 (2.45-7.01)	2.71 (1.35-5.43)
Antipsychotic prescription	4.07 (2.50-6.64)	3.09 (1.74-5.48)
Sedation-Agitation Scale scores		
Agitation (SAS >4)^*f*^	7.31 (3.27-16.36)	3.73 (1.50-9.29)
Over-sedation (SAS <3)^*f*^	2.74 (1.83-4.08)	1.30 (0.77-2.20)
Adverse event^*g*^	2.44 (1.12-5.34)	1.29 (0.53-3.15)
**Hospital and ICU**^***h ***^**characteristics**		
University-affiliated hospital (vs. community)	0.71 (0.51-0.99)	0.32 (0.17-0.61)
Closed ICU^*h*^ model (vs. open model)	1.39 (0.95-2.04)	0.59 (0.34-1.04)
Proportion of ventilator capable beds in the ICU^*h*^		
<25%	1	1
25-50%	0.55 (0.25-1.21)	0.99 (0.34-2.85)
51-75%	1.16 (0.52-2.56)	2.97 (1.03-8.55)
76-90%	1.86 (0.83-4.15)	8.34 (2.58-26.99)
>90%	0.63 (0.26-1.52)	1.97 (0.52-7.44)
Nurse to patient ratio ever <1:1	1.50 (1.05-2.15)	0.81 (0.51-1.30)
Province		
Ontario	1	1
Newfoundland and Labrador	1.98 (1.00-3.89)	1.55 (0.64-3.77)
Nova Scotia	0.49 (0.15-1.67)	0.37 (0.09-1.54)
New Brunswick	/	/
Prince Edward Island	2.96 (0.79-11.15)	4.23 (0.79-22.64)
Quebec	1.42 (0.93-2.18)	1.80 (0.99-3.29)
Manitoba	4.44 (1.47-13.42)	7.78 (2.13-28.46)
Saskatchewan	0.49 (0.22-1.13)	0.46 (0.15-1.42)
Alberta	0.87 (0.56-1.34)	1.31 (0.67-2.57)
British Columbia	1.38 (0.43-4.45)	1.02 (0.24-4.32)

^*a*^*OR*, odds ratio; ^*b*^psychiatric condition included documented depression, anxiety, bipolar disorder, schizophrenia; ^*c*^psychotropic drugs included: sedative, narcotics, methadone, antidepressants; ^*d*^dose expressed in midazolam equivalents (1 mg midazolam = 0.5 mg lorazepam); ^*e*^dose expressed in morphine equivalents (10 mg morphine = 2 mg hydromorphone = 0.1 mg fentanyl); ^*f*^Sedation-Agitation Scale; ^*g*^adverse events comprised deliberate or accidental device removal (endotracheal tube, intravenous lines, feeding tubes, urinary catheters) by patients or accidental removal by staff, and danger to self or others; ^*h*^ICU, intensive care unit; /, low frequency counts did not allow for more accurate estimates. APACHE II, Acute Physiology and Chronic Health Evaluation II.

**Table 3:** Factors independently associated with number of days of physical restraints

**Data point**	**Univariable**	**Multivariable**
	**IRR**^***a ***^**(95% CI)**	**IRR**^***a ***^**(95% CI)**
**Patient characteristics**		
Age	1.01 (0.99-1.04)	1.00 (0.98-1.03)
Male sex	0.87 (0.37-2.06)	0.73 (0.35-1.54)
Psychiatric condition^*b*^	1.13 (0.35-3.67)	1.27 (0.42-3.84)
Cognitive impairment (dementia)	0.17 (0.01-2.86)	0.28 (0.02-3.40)
Prior psychotropic drug use^*c*^	0.60 (0.26-1.41)	0.45 (0.19-1.06)
Smoking or alcohol consumption, habitual drug use	1.68 (0.71-3.98)	1.55 (0.73-3.27)
Patient category		
Surgical	1	1
Medical	1.48 (0.58-3.78)	1.74 (0.72-4.22)
Other	0.58 (0.19-1.72)	0.61 (0.24-1.56)
APACHE II score	0.97 (0.92-1.02)	0.97 (0.92-1.02)
**Treatment characteristics**		
Medication use per mechanical ventilation days		
Benzodiazepines (10 mg increments^*d*^)	1.11 (1.05-1.17)	1.07 (1.01-1.13)
Propofol (10 mg increments)	1.00 (1.00-1.01)	0.99 (0.99-1.00)
Opioids (10 mg increments^*e*^)	1.05 (1.00-1.01)	1.00 (0.99-1.10)
Daily sedation interruption	9.64 (4.23-21.94)	3.44 (1.46-8.10)
Sedation administration		
Intermittent use only	1	1
Continuous infusion only	3.35 (0.93-12.16)	0.87 (0.23-3.22)
Both	23.47 (5.97-92.27)	3.50 (0.88-13.89)
Antipsychotic prescription	45.10 (18.56-109.62)	15.67 (6.62-37.12)
Sedation-Agitation Scale scores		
Agitation (SAS >4)^*f*^	13.19 (4.12-42.15)	1.99 (0.63-6.27)
Over-sedation (SAS <3)^*f*^	11.04 (4.56-26.70)	2.62 (1.08-6.35)
Adverse event^*g*^	20.45 (3.98-105.14)	8.27 (2.07-33.08)
**Hospital and ICU**^*h*^**characteristics**		
University-affiliated hospital (vs. community)	1.51 (0.63-3.61)	0.46 (0.15-1.43)
Closed ICU^*h*^ model (vs. open model)	4.06 (1.34-12.26)	0.86 (0.25-3.00)
Proportion of ventilator capable beds in the ICU^*h*^		
<25%	1	1
25-50%	7.75 (0.87-69.27)	15.82 (1.65-151.84)
51-75%	1.97 (0.23-16.68)	5.99 (0.66-54.01)
76-90%	7.66 (0.92-63.51)	31.76 (3.02-334.41)
>90%	1.90 (0.16-22.47)	10.31 (0.67-157.93)
Nurse to patient ratio ever <1:1	2.73 (1.08-6.89)	1.75 (0.75-4.13)
Province		
Ontario	1	1
Newfoundland and Labrador	3.63 (0.35-38.16)	3.59 (0.34-37.48)
Nova Scotia	0.19 (0.00-14.37)	1.87 (0.04-97.96)
New Brunswick	0.53 (0.01-39.05)	0.83 (0.01-46.75)
Prince Edward Island	0.89 (0.04-21.49)	7.47 (0.42-133.77)
Quebec	2.74 (0.35-21.55)	2.19 (0.31-15.38)
Manitoba	1.40 (0.06-33.51)	8.95 (0.38-212.36)
Saskatchewan	0.44 (0.05-3.71)	1.56 (0.17-14.82)
Alberta	1.49 (0.05-47.70)	0.85 (0.04-20.32)
British Columbia	1.10 (0.16-7.64)	0.93 (0.15-5.61)

^*a*^IRR, incidence rate ratio; ^*b*^psychiatric condition included documented depression, anxiety, bipolar disorder, schizophrenia; ^*c*^psychotropic drugs included: sedative, narcotics, methadone, antidepressants; ^*d*^dose expressed in midazolam equivalents (1 mg midazolam = 0.5 mg lorazepam); ^*e*^dose expressed in morphine equivalents (10 mg morphine = 2 mg hydromorphone = 0.1 mg fentanyl); ^*f*^Sedation-Agitation Scale; ^*g*^adverse events comprised deliberate or accidental device removal (endotracheal tube, intravenous lines, feeding tubes, urinary catheters) by patients or accidental removal by staff, and danger to self or others; ^*h*^ICU, intensive care unit. APACHE II, Acute Physiology and Chronic Health Evaluation II.

Non-modifiable patient characteristics such as age, gender, APACHE II score, admission category, prior substance abuse, prior psychotropic medication, and pre-existing psychiatric condition or dementia were not associated with PR use, nor with the number of days PR was used.

## Discussion

This analysis of the I-CAN-SLEAP database describes prevalence of, and variables associated with, PR use in mechanically ventilated adults. Approximately half (53%) of the patients in our study were physically restrained at least once during the study period. We found that PR use in Canadian ICUs is frequent despite provincial legislation and national accreditation standards requiring restraint minimization to maintain patient safety and provide quality health care [16,31]. Internationally, use of PR in ICUs is highly variable with recent survey data and observational studies reporting prevalence rates between 15% and 100% [9,12,28,32-34]. The highest prevalence rates (for example, 90% or 100%) were found in single ICU settings [9,32].

The most important finding in our study is that predominantly treatment factors, as opposed to patient or hospital/ICU factors, influenced the use of PR. Treatment characteristics, specifically higher daily benzodiazepine and opioid doses, use of antipsychotics, and the use of continuous infusions of analgo-sedation were predictors of PR use. Also, as we hypothesized, SAS scores >4, representing agitation, predicted PR use. We also hypothesized that sedation minimization might increase PR use for the same reasons; yet we found that higher daily opiate and benzodiazepine doses were associated with PR use. We postulate that agitated patients received more medications, in combination with PR, to manage their symptoms. Our data are comparable to previous research suggesting that benzodiazepine use is more frequent in restrained patients compared to non-PR patients [21]. Antipsychotic drugs were more frequently administered to PR patients and were associated with prolonged PR use, similarly to previous findings [21]. Patients with antipsychotic prescription have a 16-fold greater number of restraint days than those without antipsychotic prescription. As antipsychotic drugs are commonly administered for delirium, they may have been a proxy for hyperactive delirium in this study. Some reports have identified associations between PR use and delirium in the ICU; for example, PR patients were more often found to be delirious than non-PR patients [21], a greater number of patients with delirium received PR and for longer durations than patients without delirium [35], and PR use was associated with an increased risk of delirium [7].

The current trend in sedation practice is to target light sedation levels using strategies such as DSI or nurse-driven sedation titration protocols to achieve improved clinical outcomes such as reduced length of stay [26]. A recent randomized controlled trial of protocolized sedation versus protocolized sedation plus DSI, with a light target level of sedation, found no significant differences in the prevalence of PR (79.4% vs. 76.4%, *P* = 0.46), nor in the duration of PR use (5.36 days (6.14) vs. 4.71 days (5.67), *P* = 0.56) between the two groups [28]. In our study, DSI was not a predictor of PR use, but was associated with a 3.4 times increase in the number of days of PR use. Although we did not seek the reasons for restraint application, we hypothesize that agitation and treatment interference were anticipated by nurses for patients undergoing DSI, a concern which has been previously reported [36]. Similarly, in our study, agitation was associated with an increased risk of PR use. Conversely, over-sedation was associated with a longer duration of PR use, suggesting failure to discontinue PR when it may no longer be justified.

Adverse events such as self-extubation were not associated with PR use in this study, but were associated with the number of days of PR use. Several cohort studies have identified the failure to use PRs as contributing to self-extubation [37-39]. However, other studies have not found PR use associated with less self-extubation. A recent systematic review of unplanned extubation in the ICU found between 25% and 87% of patients were physically restrained at time of unplanned extubation [40]. Further, one case-control study identified use of PR as associated with an increased risk of self-extubation (OR 3.1, 95% CI 1.71 to 5.70) [6]. Patients from university-affiliated hospitals were less likely to be restrained, and restrained for shorter durations. University-affiliated hospitals may use PRs less often if the clinicians working in these hospitals are more familiar with evidence-based practices or have restraint reduction protocols in place. Low nurse-patient ratios were previously described as potentially increasing PR use [9], but we found no association of PR use with nurse-patient ratio. However, this may be due to the maintenance of one-to-one nurse-patient ratios for most patient days in our study, contrasting with the heterogenous (from 1:1 to 1:4) and on average lower nurse-patient ratios reported in European centers [9].

Our study has limitations. Data collectors were not provided with a definition of PR, and as such, we cannot ascertain whether devices such as splints, intravenous arm boards, or mittens were considered as PR. PR use was recorded only once daily as a binary variable; and duration of PR use (from initiation to discontinuation) was not captured. Therefore, occurrence of more than one episode of PR in a single day was not recorded. We cannot establish the temporal relationship between risk factors and PR use. For example, future studies should aim to determine the directionality of the relationship between delirium and PR (that is, whether delirium leads to PR use or whether PR use contributes to the development of delirium) or if the relationship is bidirectional.

Additionally, we are unable to address the confounding of sedative drugs and PR. Sedatives and analgesics are used to treat agitation, anxiety, and pain in the ICU patient, but are also considered as chemical restraints, used concurrently with or alternatively to PRs. As such, future observational studies prospectively designed to explore whether use of sedative or analgesic drugs first contribute to agitation requiring use of PR or vice versa would be valuable. While we recorded the use of delirium scales, we did not record positive delirium screening. We do not know which hospitals or ICUs in our study had PR policies and protocols in place. Previous studies found that organizational or unit restraint policies and protocols may influence PR use [41,42].

Strengths of our study include the large sample size, multicenter and national representation, and a heterogeneous sample of ICUs and patients based on broad inclusion criteria, which enhance the generalizability of our data. Furthermore, data were collected prospectively, and did not rely on retrospective chart review or clinicians’ perceptions. Finally, to our knowledge, this is the first study examining predictors of PR use and number of days of use in the ICU.

## Conclusions

PR use in Canadian ICUs is common, despite legislation and guidelines to minimize use. We found that treatment characteristics specifically use of benzodiazepines, opioids, and antipsychotics, agitation, heavy sedation, sedation administration method, DSI, and occurrence of an adverse event were associated with PR use or the number of days of PR use. Understanding predictors of PR use in the ICU may increase awareness of patients at risk of receiving restraints, and enable researchers to tailor future interventions to reduce modifiable use.

## Key messages

• We found that 53% of patients in the I-CAN-SLEAP study were restrained.

• Physical restraint use in Canadian ICUs is common despite guidelines to minimize use.

• This study adds to the body of literature on the subject of physical restraint by examining predictors of use.

• Treatment characteristics that influence sedation and agitation were predominantly associated with physical restraint use and number of days of use.

## Abbreviations

APACHE II: Acute Physiology and Chronic Health Evaluation; CI: confidence interval; DSI: daily sedation interruption; ICU: intensive care unit; IRR: incidence rate ratio; MV: mechanically ventilated; OR: odds ratio; PR: physical restraint; SAS: Sedation-Agitation Scale; SD: standard deviation.

## Competing interests

The authors declare that they have no competing interests.

## Authors’ contributions

EL designed the analysis, interpreted data, and drafted the manuscript. BS designed the analysis, interpreted data, and drafted the manuscript. LR designed the study, interpreted data, and revised the manuscript critically for important intellectual content. MMP designed the study, acquired data, and revised the manuscript critically for important intellectual content. DRW designed the study, acquired data, and revised the manuscript critically for important intellectual content. SM conceived and designed the study, and revised the manuscript critically for important intellectual content. DJC designed the study and revised the manuscript critically for important intellectual content. SCL managed data and revised the manuscript critically for important intellectual content. LB conceived and designed the study, acquired and interpreted data, and revised the manuscript critically for important intellectual content. All authors read and approved the final manuscript.

## Supplementary Material

Additional file 1 **Research Ethics Boards from participating sites.** The file contains a list of Research Ethics Boards (REBs) from all 51 sites that approved the study. Click here for file

Additional file 2 **Sedation scales and equivalences.** The file contains one table with conversions of sedation scoring from the Richmond Agitation-Sedation Scale and the Ramsay Sedation Scale to the Sedation-Agitation Scale (SAS). Also displayed in this table are three classifications of sedation that were determined *a priori*: heavy sedation; calm, cooperative or lightly sedated; and agitated. Click here for file
