# Supplementary material for: Predictors of physical restraint use in Canadian intensive care units
Source: Crit Care. 2014 Mar 24;18(2):R46. doi: 10.1186/cc13789 (PMC4075126; doi:10.1186/cc13789)
Supplement: Additional file 1 — Research Ethics Boards from participating sites. The file contains a list of Research Ethics Boards (REBs) from all 51 sites that approved the study. [file cc13789-S1.pdf]

Additional file 1: Research Ethics Boards from participating sites.

REBs from the following 51 sites approved our study:

1. Western Shore Regional Hospital, Corner Brook, Newfoundland and Labrador
2. Health Sciences Centre, St. John’s, Newfoundland and Labrador
3. St. Clair’s Mercy Hospital, St. John’s, Newfoundland and Labrador
4. Central Newfoundland Regional Hospital, Grand Falls, Newfoundland and Labrador
5. Cape Breton Island Hospital, Cape Breton, Nova Scotia
6. Truro Regional Hospital, Truro, Nova Scotia
7. Queen Elizabeth Hospital, Charlottetown, Prince Edward Island
8. Atlantic Health Sciences Corporation, Saint John Regional Hospital, Saint John, New Brunswick
9. South-East Regional Health Authority, Moncton, New Brunswick
10. Hôpital Cité de la Santé de Laval, Quebec
11. MUHC, Hopital Royal Victoria, Montreal, Quebec
12. MUHC, Montreal General Hospital, Montreal, Quebec
13. Hopital Sacré-Coeur, Montreal, Quebec
14. Hopital Enfant Jésus, Montreal, Quebec
15. Hotel Dieu de Quebec, Quebec
16. CHUM - Hotel Dieu de Montreal, Quebec
17. CHUS -Sherbrooke, Quebec
18. Mount Sinai Hospital, Toronto, Ontario
19. Toronto Western Hospital, University Health Network, Toronto, Ontario
20. Toronto General Hospital, University Health Network, Toronto, Ontario
21. Hamilton Health Sciences – ICU West, Hamilton, Ontario
22. Hamilton Health Sciences – ICU East, Hamilton, Ontario
23. Hamilton Health Sciences McMaster University Medical Centre, Hamilton, Ontario
24. St. Joseph’s Healthcare, Hamilton, Ontario
25. Henderson Hospital, Hamilton, Ontario
26. Queensway Carleton, Ottawa, Ontario
27. St. Mary’s General Hospital, Kitchner, Ontario
28. Trillium Hospital, Mississauga, Ontario
29. Sault Area Hospital, Sault St. Marie, Ontario
30. Hotel Dieu Grace Hospital, Windsor, Ontario
31. Lakeridge Hospital, Oshawa, Ontario
32. Toronto East General Hospital, Toronto, Ontario
33. Royal Victoria Hospital, Barrie, Ontario
34. Rouge Valley Hospital, Ajax, Ontario
35. Markham Stouffville Hospital, Markham, Ontario
36. William Osler Health Centre, Brampton, Ontario
37. London Health Sciences Centre, London, Ontario
38. Health Sciences Centre, Winnipeg, Manitoba
39. Grace Hospital, Winnipeg, Manitoba
40. Brandon Regional Health Authority, Brandon, Manitoba
41. Foothills Hospital, Calgary, Alberta
42. Rockycrest Hospital, Calgary, Alberta
43. Capital Health, University of Alberta Medical Centre, Edmonton, Alberta
44. Caritas Health Group, University of Alberta Medical Centre, Edmonton, Alberta
45. Grey Nuns Hospital, Edmonton, Alberta
46. Regina Qu’Appelle Health Region, MICU, Regina, Saskatchewan

47. Regina Qu’Appelle Health Region, Regina, Saskatchewan
48. Fraser Health Authority, Chilliwack General Hospital, Chilliwack, British Columbia
49. Fraser Health Authority, Surrey Memorial Hospital, British Columbia
50. Burnaby Hospital, Burnaby, British Columbia
51. Langley Memorial Hospital, Langley, British Columbia
