# Supplementary material for: Predictors of physical restraint use in Canadian intensive care units
Source: Crit Care. 2014 Mar 24;18(2):R46. doi: 10.1186/cc13789 (PMC4075126; doi:10.1186/cc13789)
Supplement: Additional file 2 — Sedation scales and equivalences. The file contains one table with conversions of sedation scoring from the Richmond Agitation-Sedation Scale and the Ramsay Sedation Scale to the Sedation-Agitation Scale (SAS). Also displayed in this table are three classifications of sedation that were determined a priori: heavy sedation; calm, cooperative or lightly sedated; and agitated. [file cc13789-S2.pdf]

Additional file 2: Sedations scales and equivalences.

	Sedation-Agitation Scale[a]	Richmond Agitation-Sedation Scale[b]	Ramsay Sedation Scale[c]
Heavy sedation<br>(SAS <3)	1 Unarousable.	-5 Unarousable.	6 Asleep, no response to light glabellar tap or loud noise.
	2 Very sedated.	-4 Deep sedation.	5 Asleep, sluggish response to light glabellar tap or loud noise stimulus but no response to painful stimulus.
Calm, cooperative or lightly sedated<br><br>(SAS 3-4)	3 Sedated.	-3 Moderate sedation.	4 Asleep, brisk response to light glabellar tap or loud noise.
		-2 Light sedation.<br>-1 Drowsy.	3 Awake, responds only to commands.
	4 Calm, cooperative.	0 Alert and calm.	2 Awake, cooperative, accepting ventilation, oriented, tranquil.
Agitated<br>(SAS >4)	5 Agitated.<br>6 Very agitated.<br>7 Dangerous agitation.	+1 Restless.<br>+2 Agitated.<br>+3 Very agitated.<br>+4 Combative.	1 Awake, and anxious, agitated, or restless.

[a] Riker RR, Picard JT, Fraser GL: **Prospective evaluation of the Sedation-Agitation Scale for adult critically ill patients**. *Crit Care Med* 1999, **27**:1325-1329.
[b] Sessler CN, Gosnell MS, Grap MJ, Brophy GM, O'Neal PV, Keane KA, Tesoro EP, Elswick RK: **The Richmond Agitation-Sedation Scale: validity and reliability in adult intensive care unit patients**. *Am J Respir Crit Care Med* 2002, **166**:1338-1344.
[c] Ramsay MA, Savege TM, Simpson BR, Goodwin R: **Controlled sedation with alphaxalone-alphadolone**. *Br Med J* 1974, **2**:656-659.
